# Communication skills in medicine: where do we come from and where are we going?

**DOI:** 10.3325/cmj.2015.56.311

**Published:** 2015-06

**Authors:** Guillermo Ferreira-Padilla, Teresa Ferrández-Antón, José Baleriola-Júlvez, Marijana Braš, Veljko Đorđević

**Affiliations:** 1 ComunVista Research Group of the Catholic University of Valencia, Valencia, Spain *g.ferreirapadilla@gmail.com*; 2 Primary Care Center “La Vall D’Uixó,” Castellón, Spain; 3 Centre for Palliative Medicine, Medical Ethics and Communication Skills, School of Medicine University of Zagreb, Zagreb, Croatia

The physician-patient relationship has changed throughout history, as the role of physician has been transformed. Modern physicians need to be educated on how to use highly specialized knowledge when approaching the patient as a unique and whole person living in a given psychological, social, and material context. This relationship evolved from a paternalistic model to a cooperative-deliberative one, representing a meeting between two “experts:” the physician as the medical expert and the patient as the expert on himself. According to this model, communication between patients and physicians must be based on common understanding in a caring and dynamic relationship that also involves the patient’s family. Nevertheless, this new approach is not free from difficulties, because physicians have to learn to adapt to its demands. This situation has led to a new concept known as the “empowered patient” (1).

Effective physician-patient communication is a central clinical function in building a therapeutic relationship. Therefore, in recent decades great attention has been paid to the quality of communication in medicine. However, the educational background and characteristics of communication skills teaching are a less studied field. Anglo-Saxon countries are pioneers in integrating this subject into undergraduate and postgraduate medical education. Less is known about other countries, especially Spanish-speaking and Central and Eastern European countries (2-4). The only way the future physicians (today’s students) can develop effective communication with their patients is to integrate this teaching at the university level. Therefore, it was necessary to analyze how teaching of communication skills in medical schools has evolved. This essay provides a brief historical analysis of the integration of this teaching in the pioneering countries. We also focus our attention to Spain and Croatia, where our teams come from. It can be said that these reflections are the result of teamwork and collaboration between Croatia and Spain.

## The seventies: difficult beginnings

This decade marked a turning-point in communication skills teaching. In this period, the essential clinical skills of physicians were based solely on clinical and technical knowledge, physical examination and resolution of medical problems (4). There were no communication courses. In fact, most doctors believed that communication was an innate skill that could not be learned (2,4). However, a new education method began to develop based on training in communication skills and the clinical interview through closed-circuit television techniques (5).

## The eighties: first recommendations

In the eighties, early publications supporting education in communication started to emerge. In the United Kingdom, the General Medical Council reported that medical students should be taught to communicate clearly, sensitively, and effectively with patients (6). Since then, this type of education has become common in the undergraduate medical curricula of this country. A short time later, communication skills courses started being taught in several medical schools of the United States (7,8).

## The nineties: communication skills can and should be taught

Since the beginning of the nineties, numerous reports recognized the importance and the need for including this content in the undergraduate curricula (7-9). For example, Simpson et al stated that communication skills can and should be taught (9). The integration of this competence into medical schools of the United States was favored by the Association of American Medical Colleges. Its report entitled: “Contemporary Issues in Medicine: Communication in Medicine” (10) concluded that all medical schools must formally assess the quality of communication of their students (10). This was a Copernican turn from what had been taught in medical schools until then. However, Hulsman et al reported that despite the growing interest in teaching of communication skills in medical schools, relatively limited curricular time was spent on it (11).

At the same time, The Accreditation Council for the Graduate Medical Education Outcome Project began to focus on resident’s performance in the six competencies of medical education, including interpersonal and communication skills. More precisely, it was affirmed that residents must be able to demonstrate interpersonal and communication skills that result in effective information exchange and teaming with patients, patients’ families, and professional associates (12).

## Communication skills teaching in Spain

Initial reports about communication skills teaching in Spanish-speaking medical schools appeared relatively recently. In 1990, the Association of Medical Schools of Chile reformulated its current curriculum, giving prominence to communication skills teaching (4). A sincere interest in this competence has also been expressed in other Spanish-speaking countries, although it has not yet been fully reflected in their curricula (4). During the nineties, this content was not present in the medical schools in Spain (2,3,13,14). However, authors with excellent publications on this topic have insisted on integration of communication skills teaching into the universities’ curricula around the world (14-17). This, together with the recent changes in the European curriculum due to the Bologna Process and the evolution of communication process in the medical field led researchers to investigate the panorama of training in communication skills in Spain (2,3). ComunVista Research Group of the Catholic University of Valencia provided the first descriptive and systematic publication of the curricula development of all Spanish medical schools (2). The conclusion was that 71.43% (30 of 42) of the Spanish medical schools had integrated communication skills in their curriculum and taught communication skills as a subject. The average course credits assigned to this skills increased considerably from 1990 (0 credits, equivalent to 0 hours) to 2014 (2.77 European credits, equivalent to 76.17 hours: 26.66 classroom hours and 49.51 non-classroom hours). However, the recommendations of the National Agency for Quality Assessment and Accreditation of Spain (NAQAA) in most Spanish medical schools had not been completely established yet. For example, NAQAA proposed 5.0 European credits for this competence. According to this agency, communication skills are one of the seven subject areas that students must acquire during their medical studies in Spain (18). On the other hand, Spanish public universities seem to be more aware of this problem, and 25 of 32 public universities had communication as a subject in their curricula. In the case of private universities, this number was 5 of 10. Of these 10 private universities, 7 have religious affiliation while 3 are secular. Three of the 7 religious institutions and all secular medical schools had communication in their curricula. Furthermore, this subject was integrated in 23 of 30 medical schools in the first year, becoming a general rule by the second year. A high percentage of medical schools that integrate communication as a transversal course still exist in Spain. Fortunately, curricula designs are evolving, and this competence is being implemented in the syllabus of most Spanish autonomous communities and schools of medicine. A project on communication skills teaching in Spanish nursing schools, which was carried out together with researchers from the University of Alicante (Spain), found that all of 110 (100%) universities had communication teaching integrated in their curricula, but that it was more integrated in nursing schools than medicine schools curricula. At the time when the graduate and postgraduate teaching of communication skills was being integrated into the Anglo-Saxon schools, in the Spanish universities this process had not yet begun and these skills were taught only in postgraduate medical education (13). In this sense, the primary health care played a fundamental role (2,13). Since then, the teaching of communication skills has been integrated into the portfolio of the family and community medicine residents and added gradually as a part of the general common training and studies, and now it is present in other medical specialties and other fields inside health science.

## Communication skills teaching in Croatia

Croatia shows a long tradition in the promotion of person-centered medicine and people-centered health care (19,20). Although for the past twenty years communication skills have been taught in various courses in Croatian medical schools, major curricular changes have occurred in the last five years, especially at the University of Zagreb School of Medicine. In September of 2010, the Centre for Palliative Medicine, Medical Ethics, and Communication Skills (CEPAMET) was founded as a department of the University of Zagreb School of Medicine and ever since has served as a center for communication skills education for medical students, health care professionals, volunteers, and the general public (19). An elective course, Communication in Medicine, was introduced for students of the fourth, fifth, and sixth year that teaches methods of experiential learning (role-play, real patient encounters, simulated patient encounters) as well as the Calgary-Cambridge model for the medical interview. Another important novelty was the introduction of mandatory longitudinal course, Fundamentals of Medical Skills, which is taught to students from the first throughout the sixth year and is divided into clinical skills and communication skills components. This subject is taught in small groups (8-12 students per instructor), which required training of a large number of instructors. This course is also taught to students in the English-language medical program.

Communication skills teaching is a part of general physician competencies teaching, which is a mandatory part of post-graduate training for all specialists in Croatia. Special modules dedicated to communication are taught at the post-graduate specialist level, dealing with the specifics of communication in the individual specialties (eg, communication skills in psychiatry, oncology, etc). CEPAMET also organizes various post-graduate continuing education workshops dedicated to communication skills within specific clinical fields, especially in oncology and palliative medicine. Manuals and other publications have been created for each of these courses, and CEPAMET has undertaken various research projects on this subject. The medical interview provides a framework through which physicians can explore and understand patients’ concerns, fears, misconceptions, and what they bring to their illness, while taking into consideration their culture, the availability of various treatment options, and financial considerations. The Zagreb model of person-centered medical interview is focused not only on the disease but on patient’s quality of life in the context of health and disease. Person-centered medical interview is an important bridge between personalized and person-centered medicine (21). When we talk about different forms of communication in medicine, we must never forget the importance of communication through art. Art should be used as a therapeutic technique, as well as means of raising public awareness of some medical problem. CEPAMET has also pioneered many projects related to art as one of the best forms of educating medical professionals and others involved in treatment and decision-making (21).

Croatia has begun the serious task of incorporating communication skills education and recognizing it as an exceptionally important component of medical education, post-graduate specialty training, and continuing education. Although the numerous positive steps undertaken on this subject are already evident with regard to attitudes, knowledge, and skills, this is merely the beginning of a long journey that will require numerous research projects and continued development of educational programs, especially considering the local culture and traditions. It is especially important to mention that, within its educational programs in communication skills, Croatia emphasizes the importance of person-centered medicine and the culture of health (22,23).

## Conclusion

Communication in medicine is considered as a fundamental clinical skill to establish a relationship with the patient, paving a way to successful diagnosis and treatment. Communication skills training is internationally accepted as an essential component of medical education. Since communication skills can be learned and mastered by practice, experiential learning is important, and individualized and interactive format of teaching should be applied adhering to the principles of evidence-based and person-centered medicine. Human relationship is what matters most!
